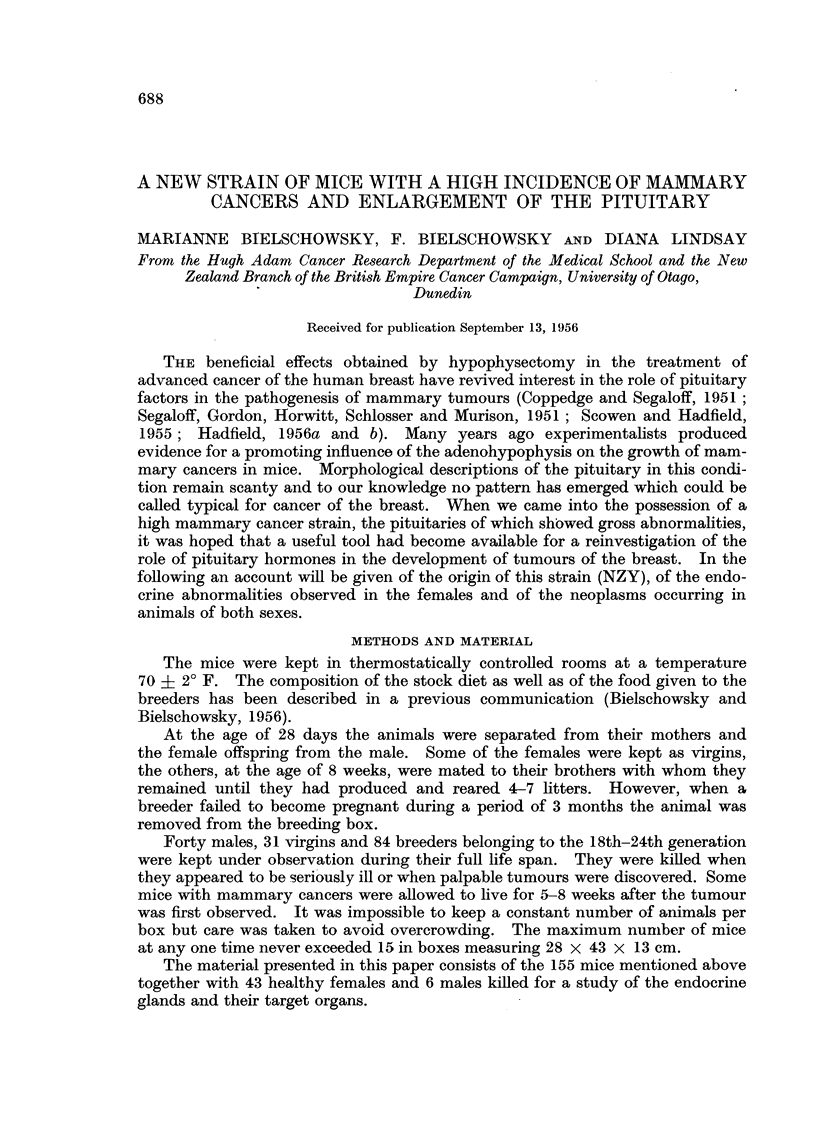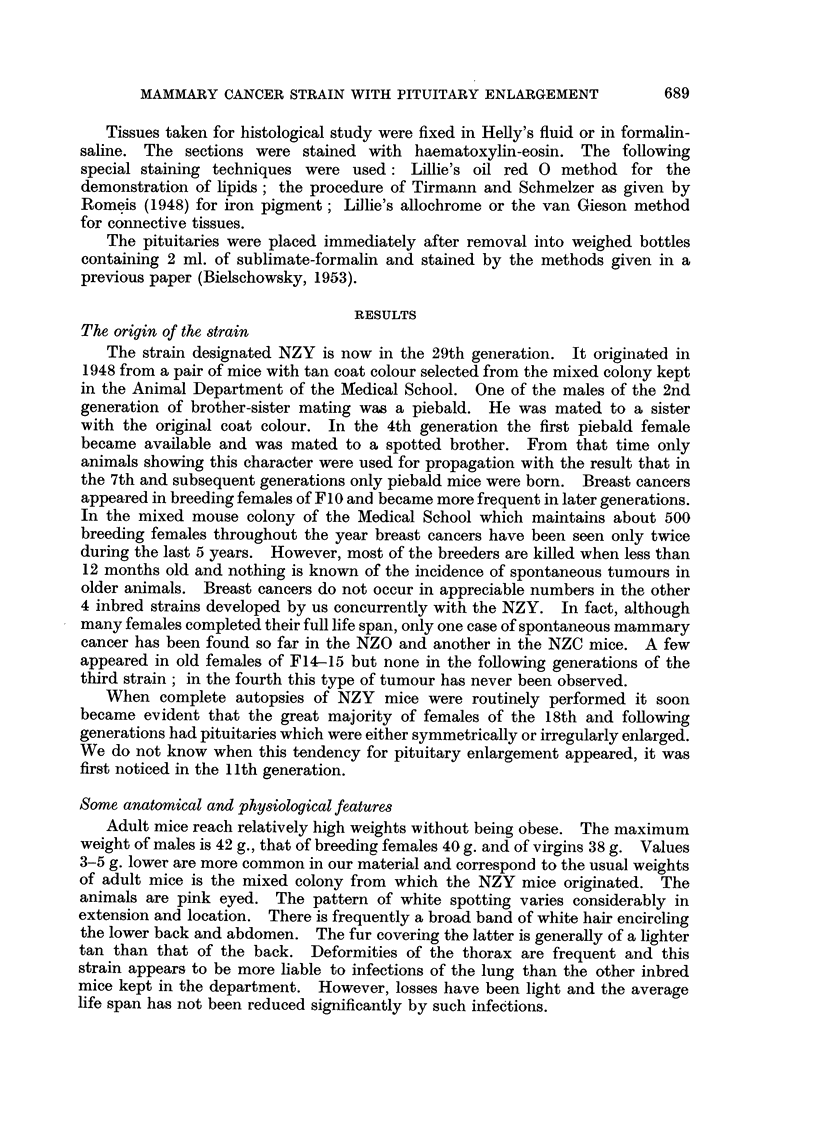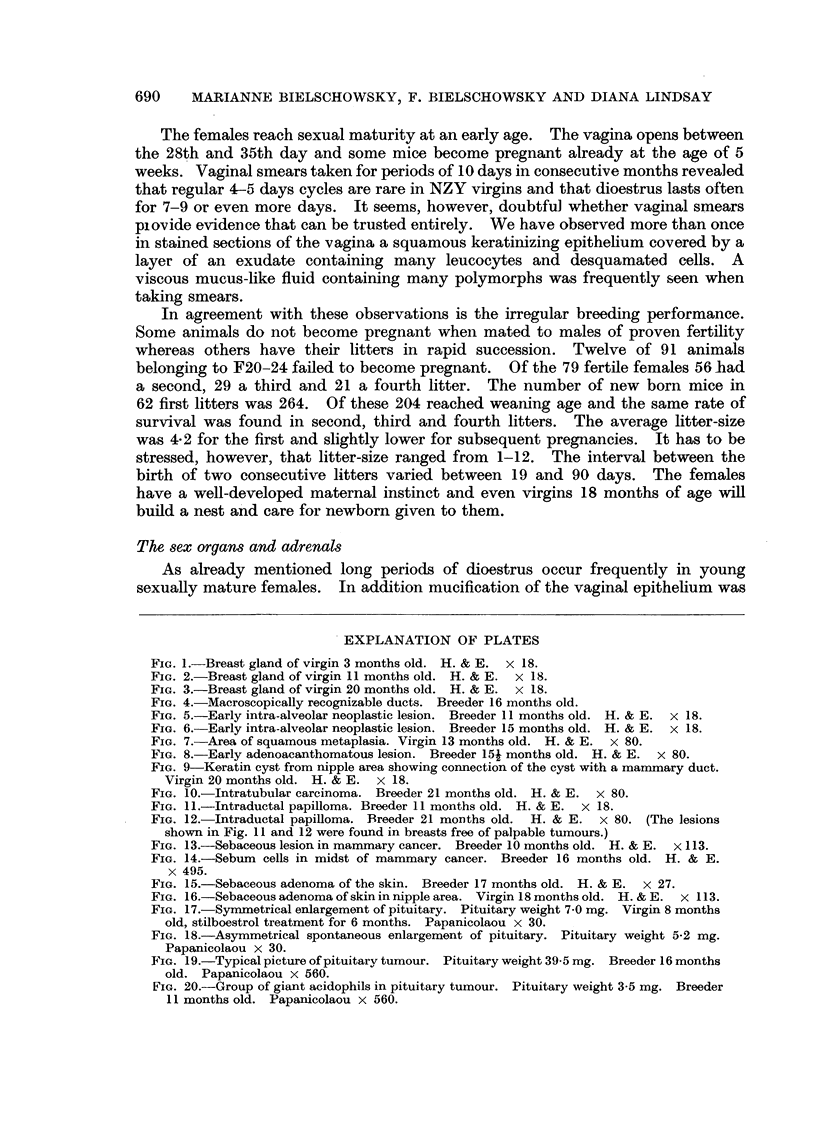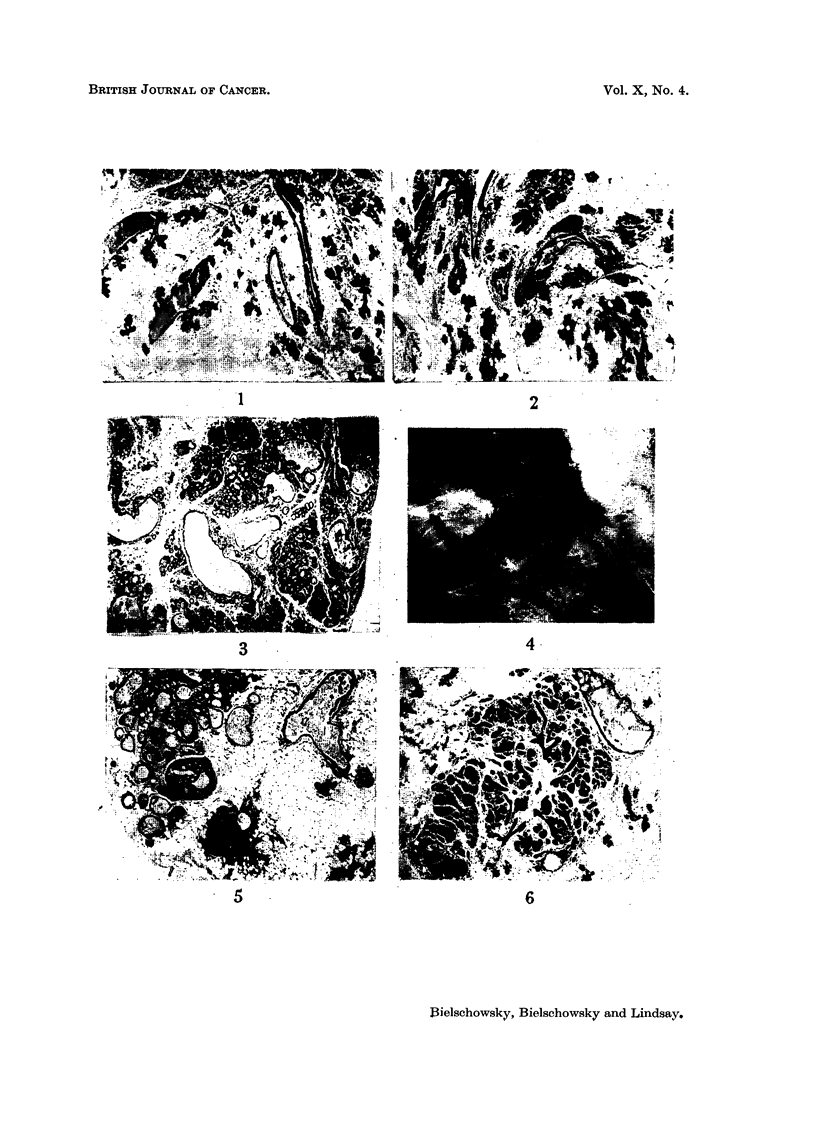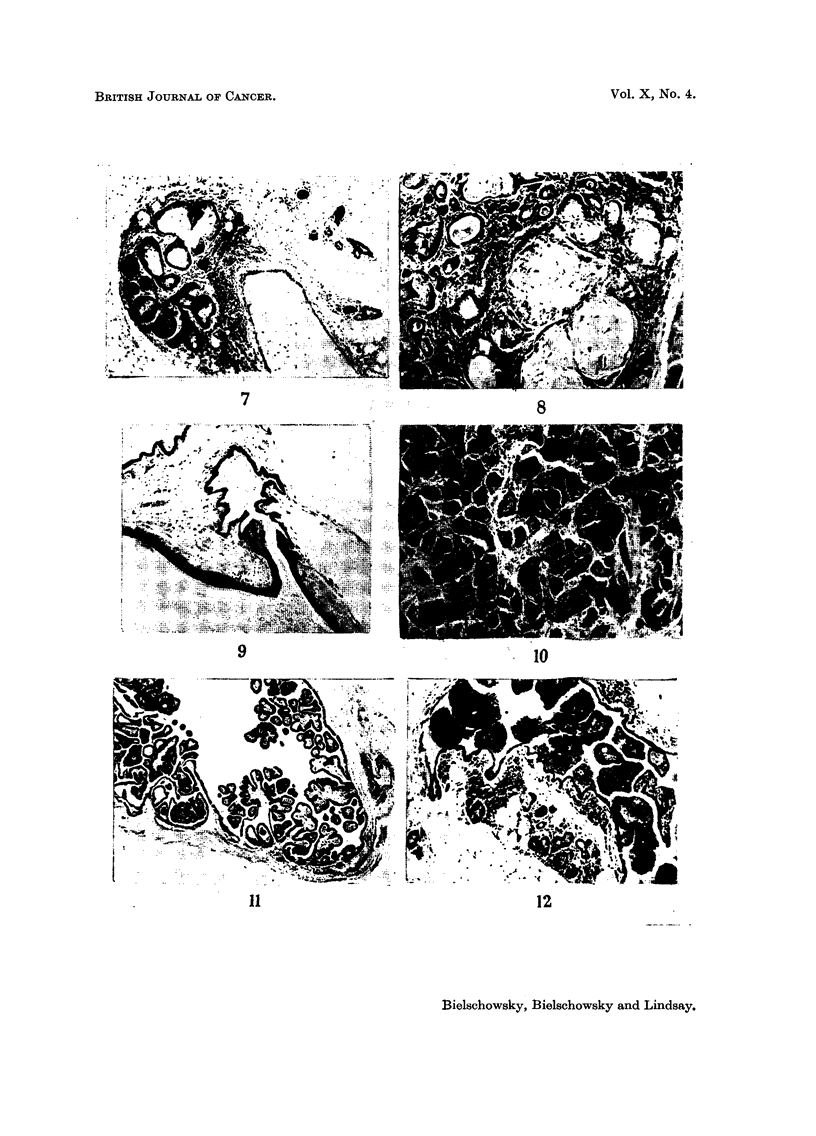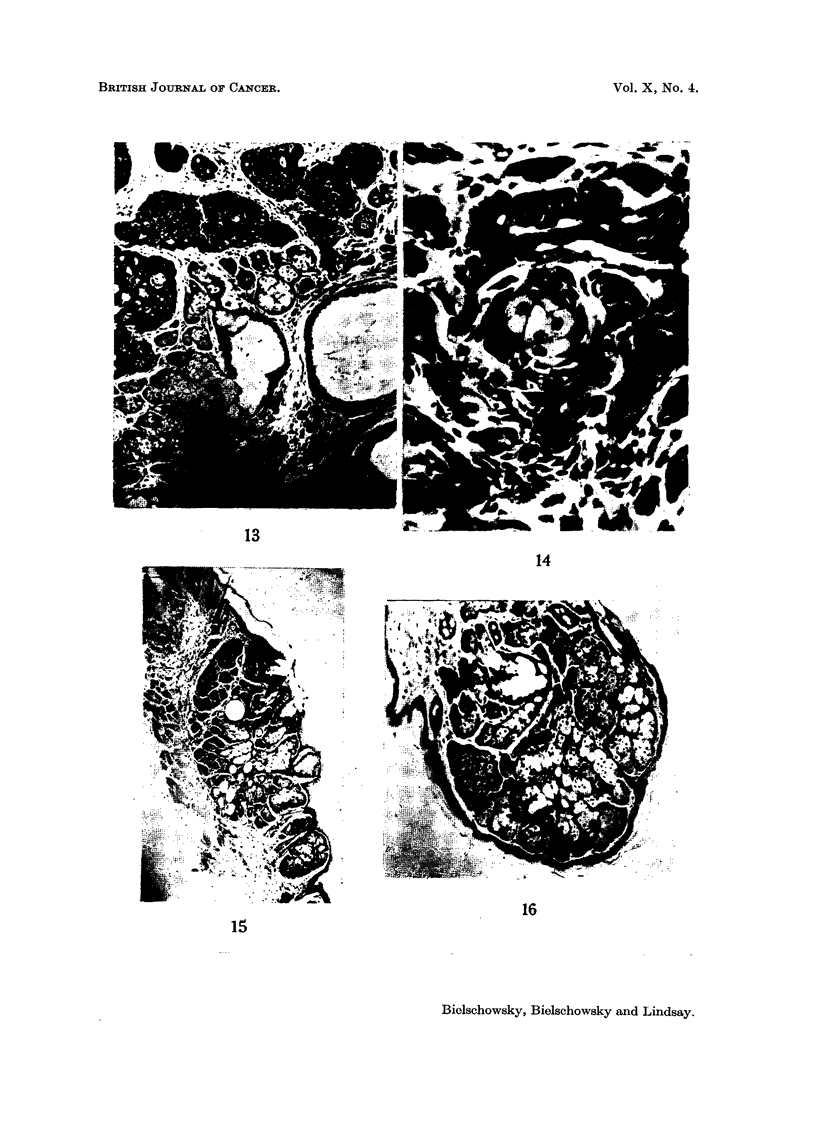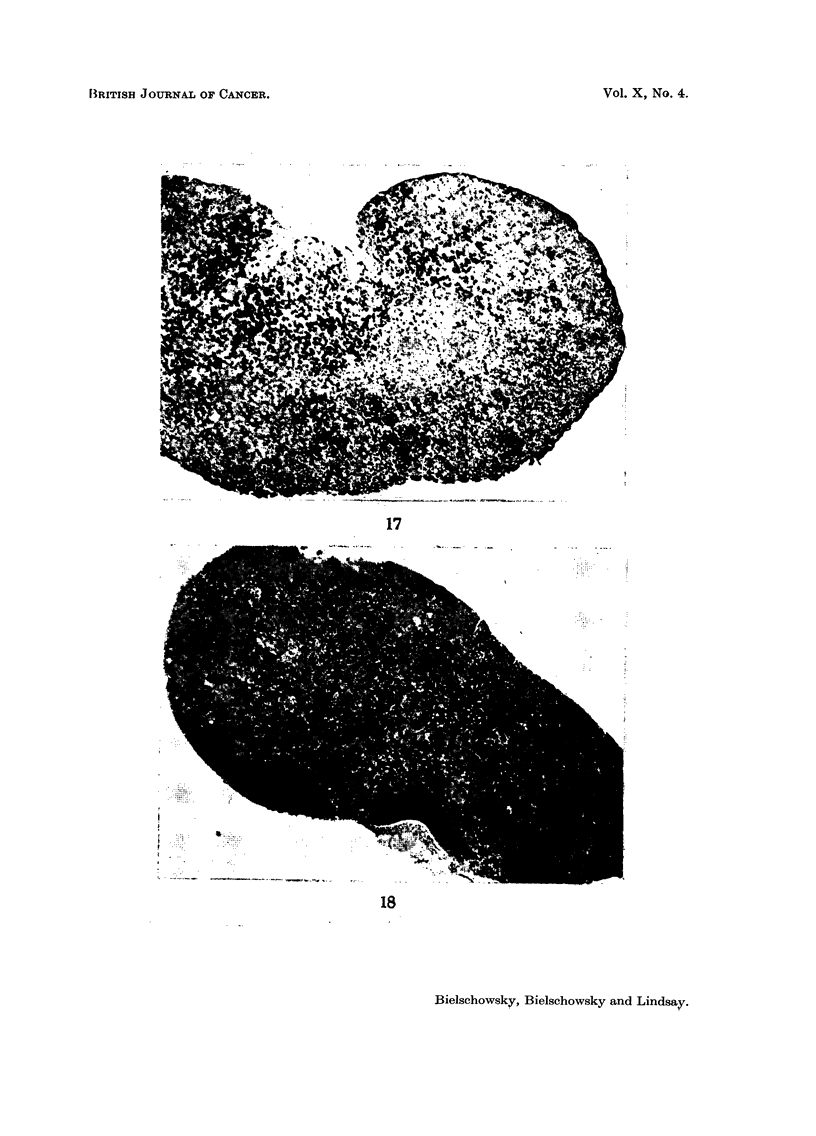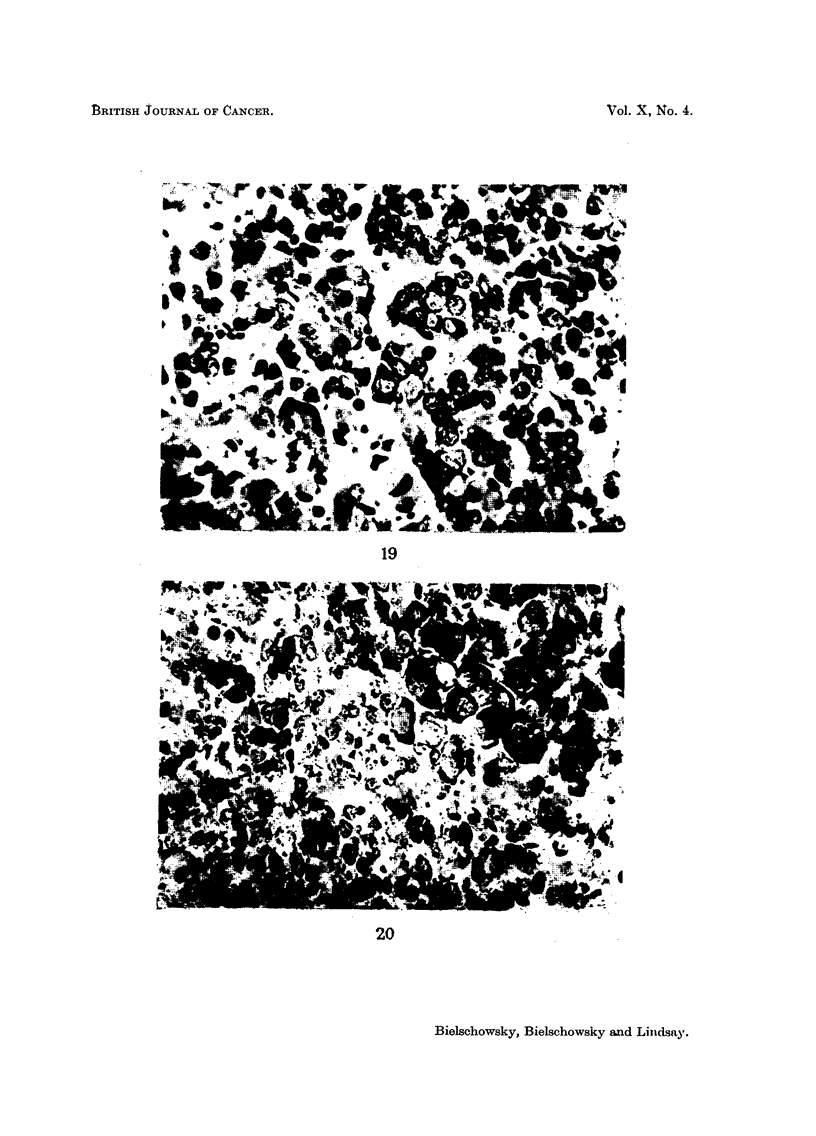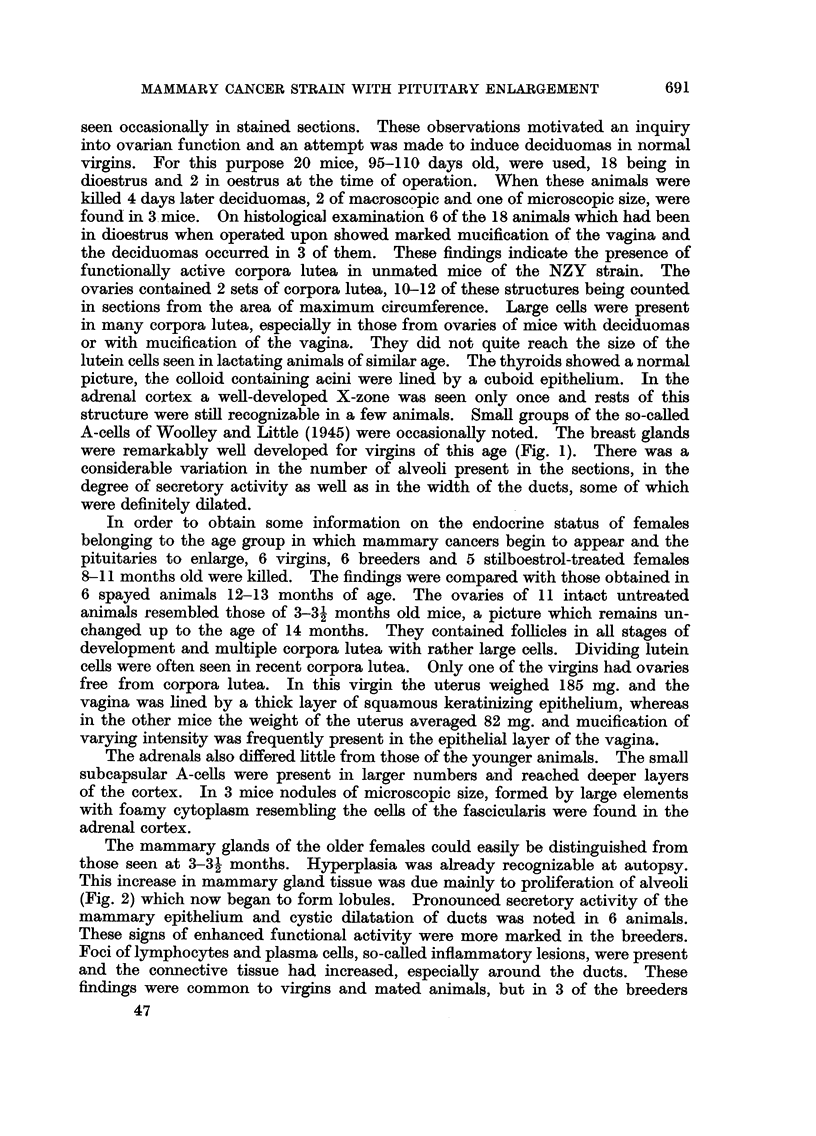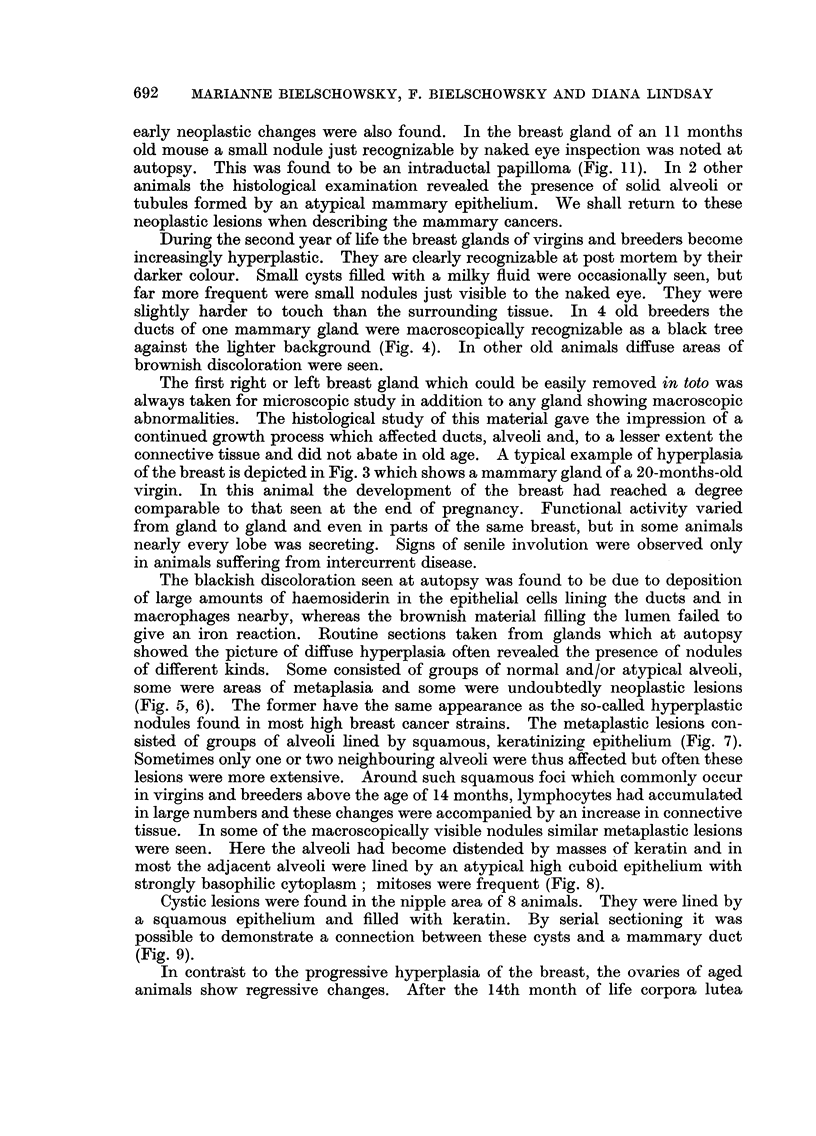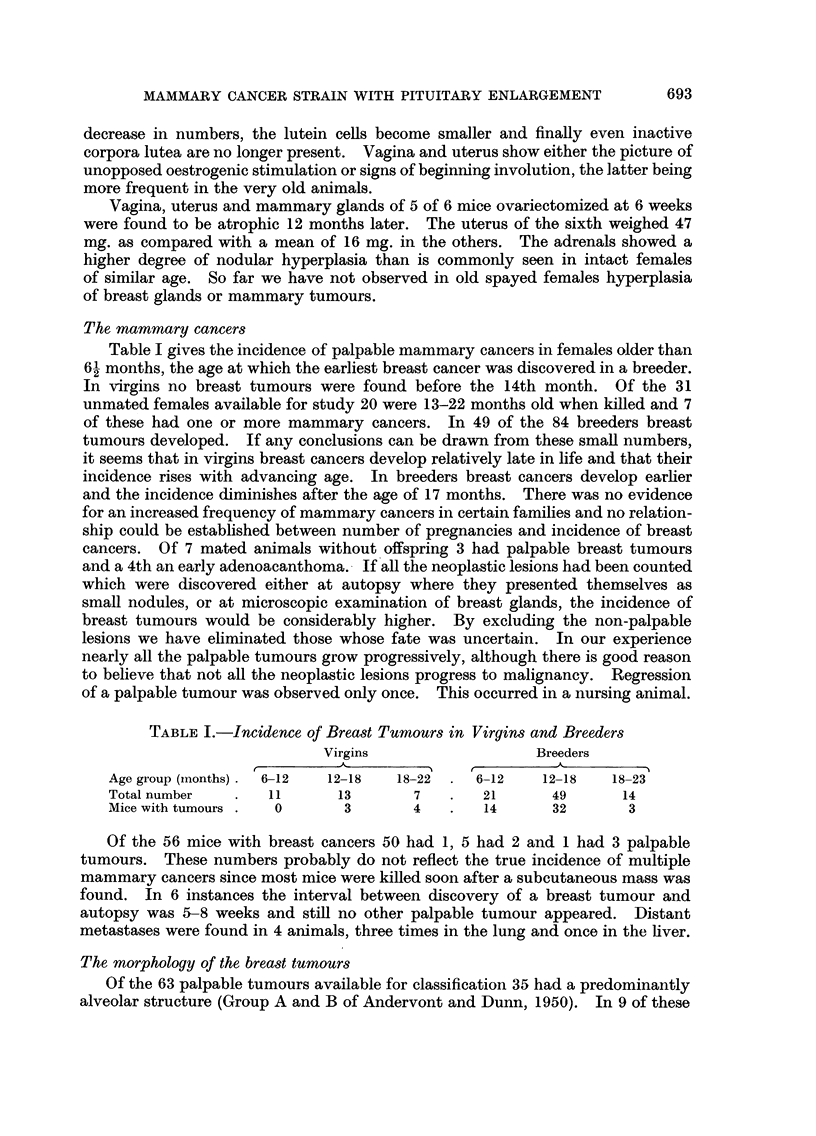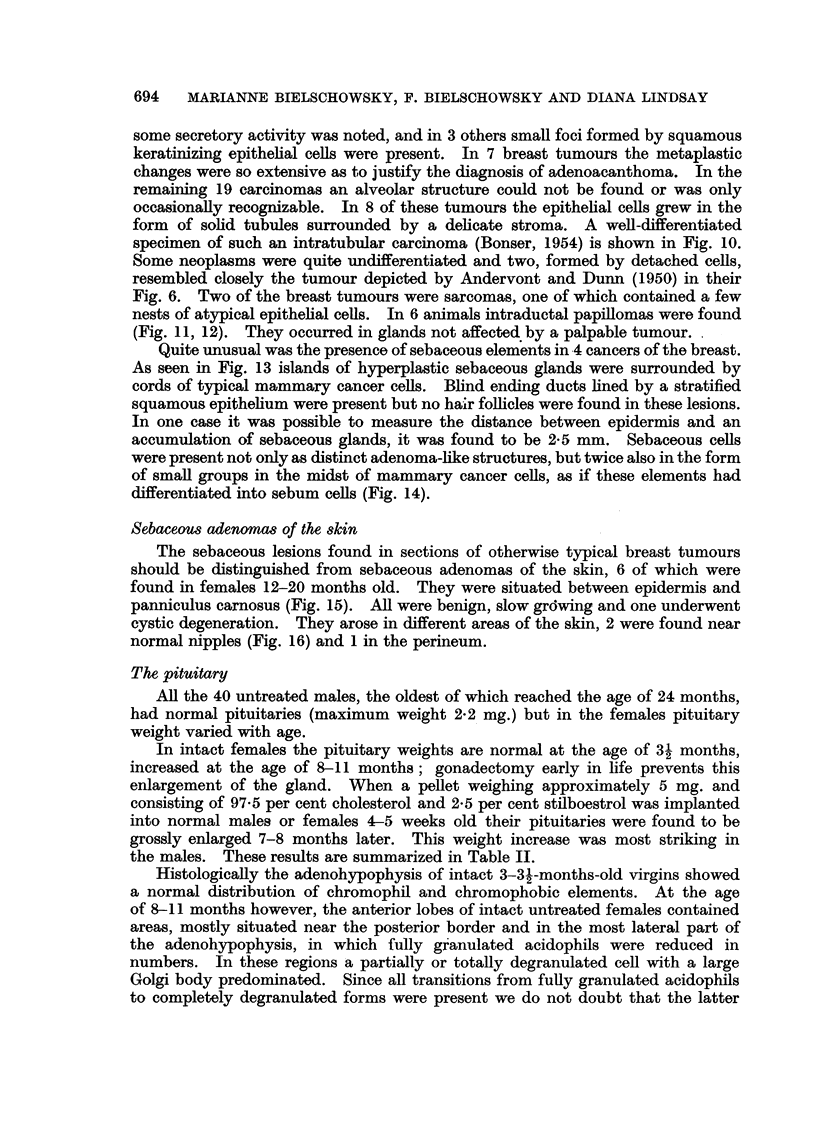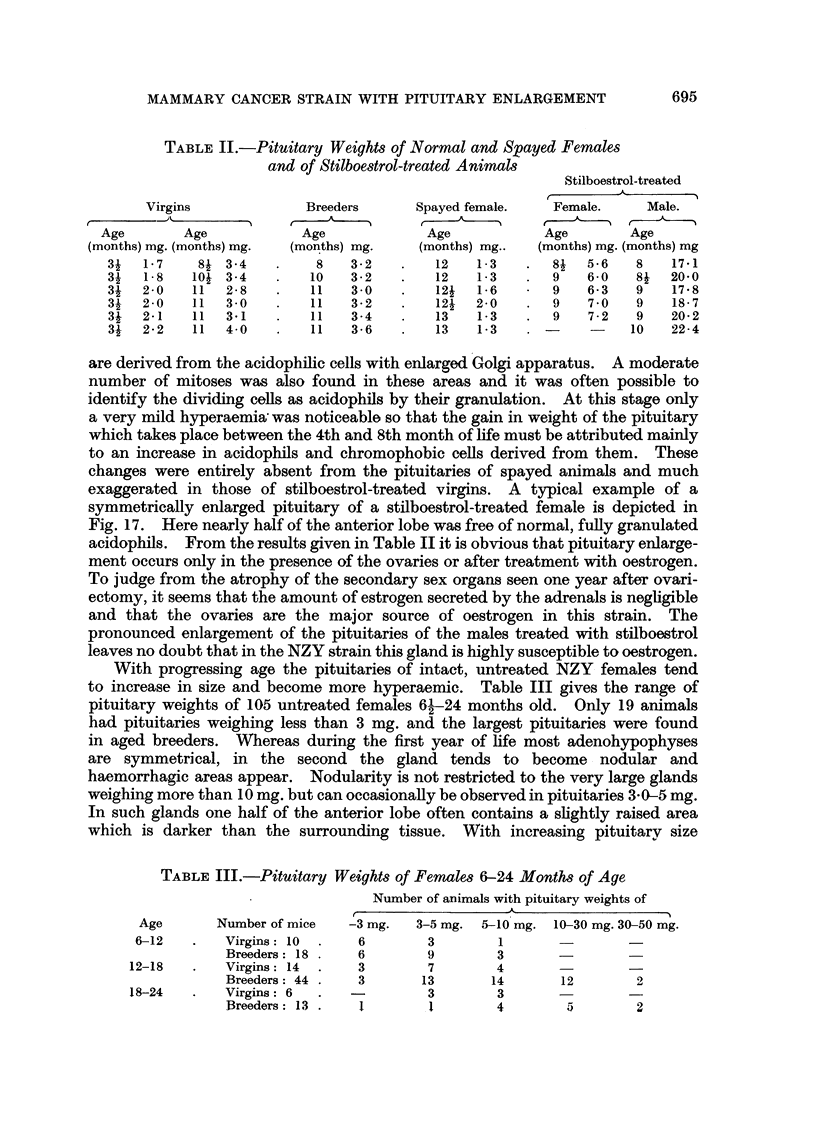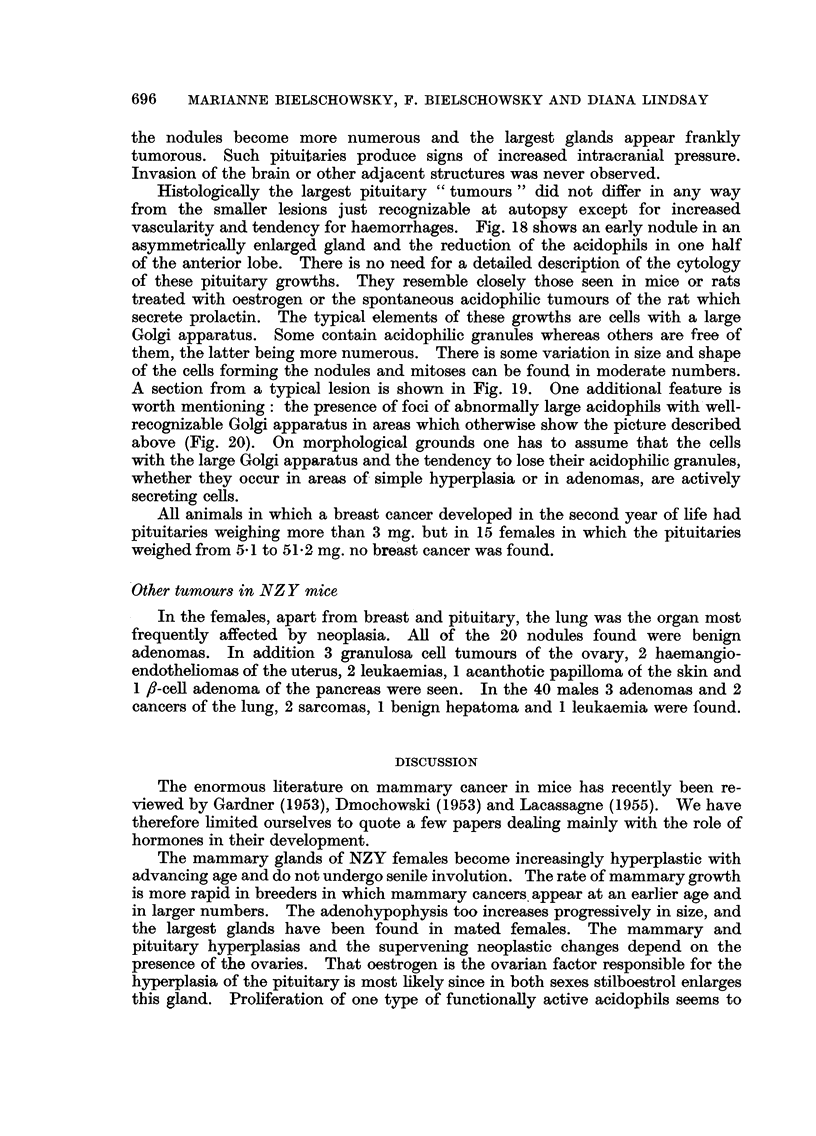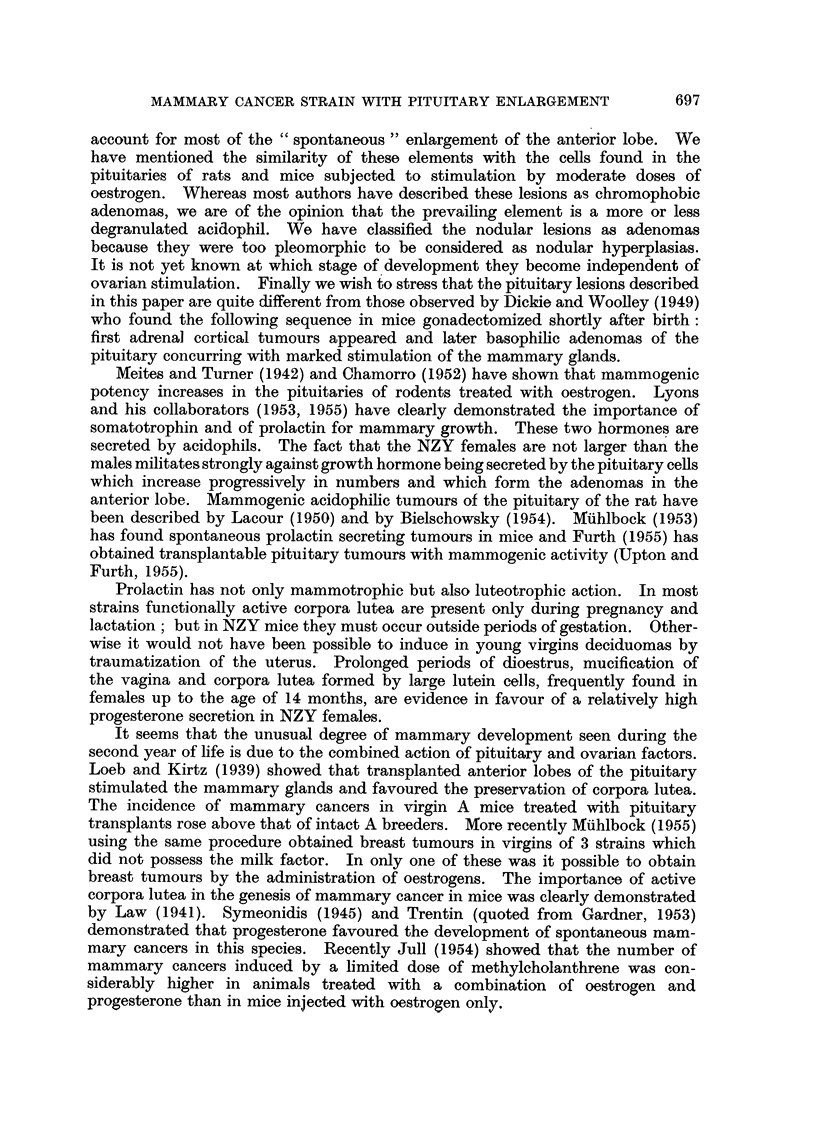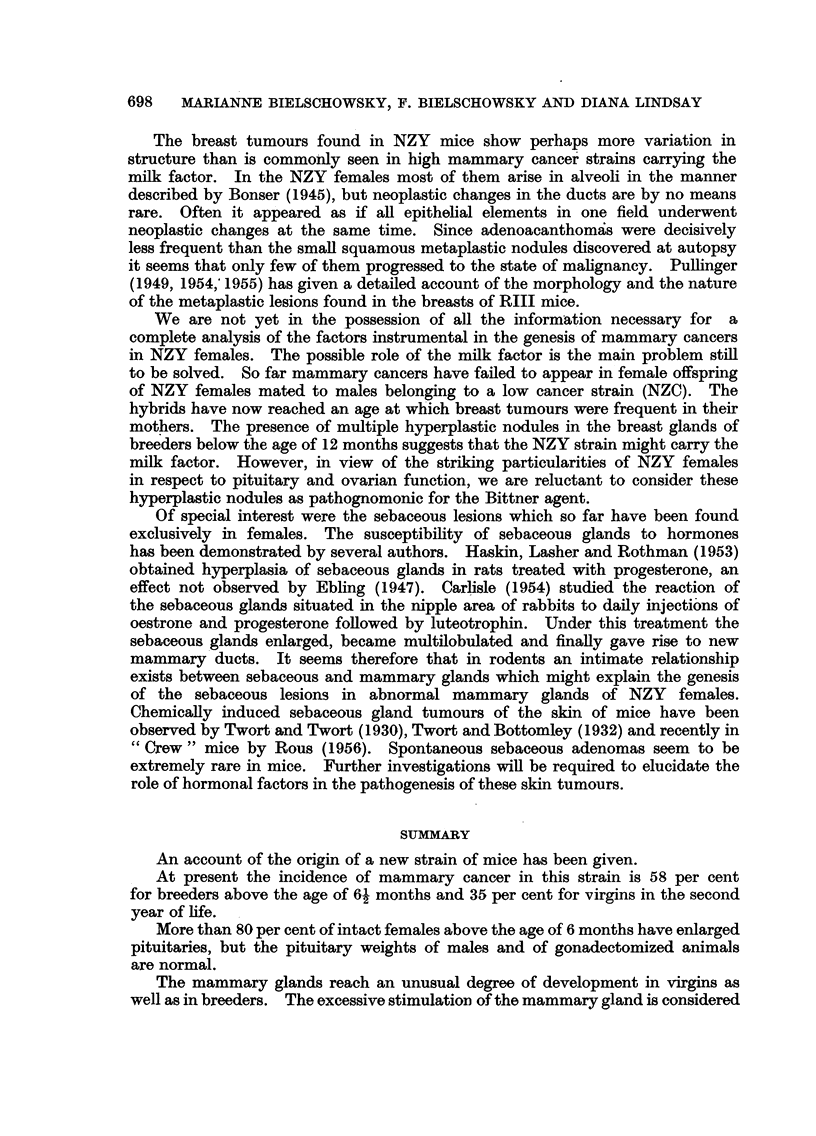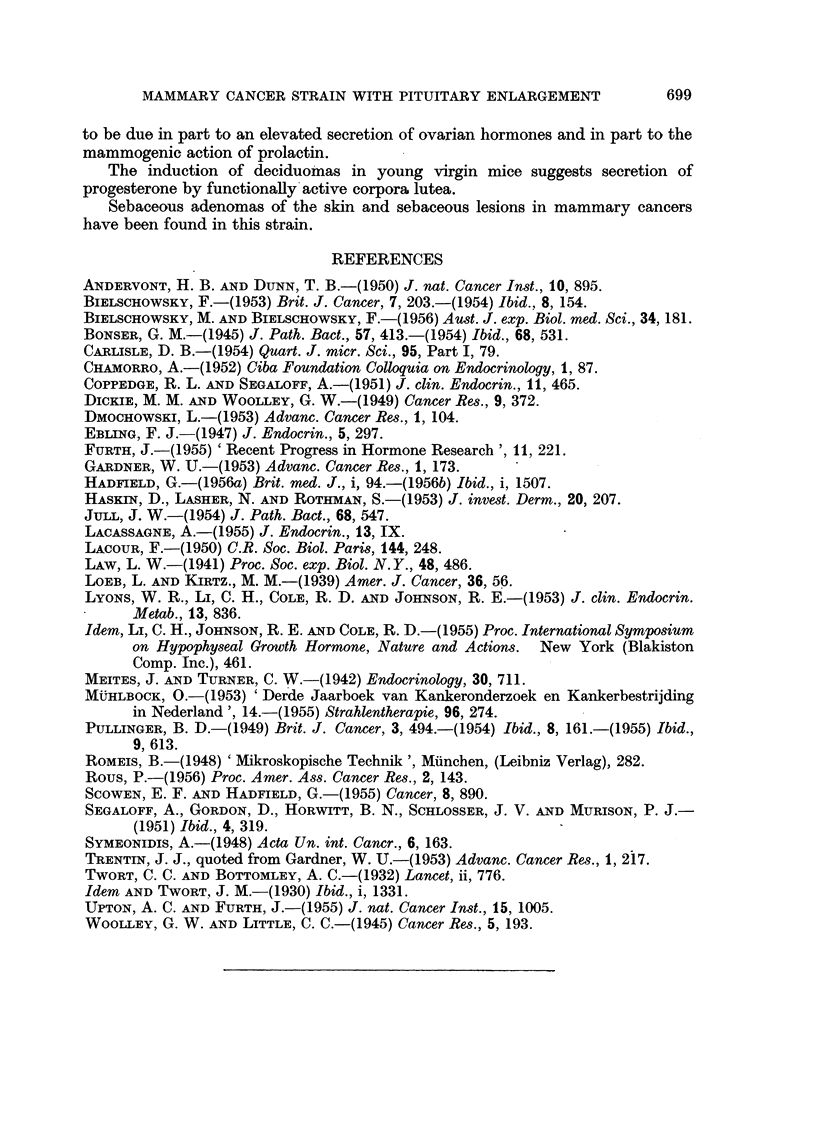# A New Strain of Mice with a High Incidence of Mammary Cancers and Enlargement of the Pituitary

**DOI:** 10.1038/bjc.1956.83

**Published:** 1956-12

**Authors:** Marianne Bielschowsky, F. Bielschowsky, Diana Lindsay

## Abstract

**Images:**


### A NEW STRAIN OF MICE WITH A HIGH INCIDENCE OF MAMMARY CANCERS AND ENLARGEMENT OF THE PITUITARY

MARIANNE BIELSCHOWSKY, F. BIELSCHOWSKY AND DIANA LINDSAY

*From the Hugh Adam Cancer Research Department of the Medical School and the New Zealand Branch of the British Empire Cancer Campaign, University of Otago, Dunedin*

Received for publication September 13, 1956

THE beneficial effects obtained by hypophysectomy in the treatment of advanced cancer of the human breast have revived interest in the role of pituitary factors in the pathogenesis of mammary tumours (Coppedge and Segaloff, 1951; Segaloff, Gordon, Horwitt, Schlosser and Murison, 1951; Scowen and Hadfield, 1955; Hadfield, 1956*a* and *b*). Many years ago experimentalists produced evidence for a promoting influence of the adenohypophysis on the growth of mammary cancers in mice. Morphological descriptions of the pituitary in this condition remain scanty and to our knowledge no pattern has emerged which could be called typical for cancer of the breast. When we came into the possession of a high mammary cancer strain, the pituitaries of which showed gross abnormalities, it was hoped that a useful tool had become available for a reinvestigation of the role of pituitary hormones in the development of tumours of the breast. In the following an account will be given of the origin of this strain (NZY), of the endocrine abnormalities observed in the females and of the neoplasms occurring in animals of both sexes.

### METHODS AND MATERIAL

The mice were kept in thermostatically controlled rooms at a temperature $70 \pm 2°$ F. The composition of the stock diet as well as of the food given to the breeders has been described in a previous communication (Bielschowsky and Bielschowsky, 1956).

At the age of 28 days the animals were separated from their mothers and the female offspring from the male. Some of the females were kept as virgins, the others, at the age of 8 weeks, were mated to their brothers with whom they remained until they had produced and reared 4–7 litters. However, when a breeder failed to become pregnant during a period of 3 months the animal was removed from the breeding box.

Forty males, 31 virgins and 84 breeders belonging to the 18th–24th generation were kept under observation during their full life span. They were killed when they appeared to be seriously ill or when palpable tumours were discovered. Some mice with mammary cancers were allowed to live for 5–8 weeks after the tumour was first observed. It was impossible to keep a constant number of animals per box but care was taken to avoid overcrowding. The maximum number of mice at any one time never exceeded 15 in boxes measuring $28 \times 43 \times 13$ cm.

The material presented in this paper consists of the 155 mice mentioned above together with 43 healthy females and 6 males killed for a study of the endocrine glands and their target organs.

Tissues taken for histological study were fixed in Helly's fluid or in formalin-saline. The sections were stained with haematoxylin-eosin. The following special staining techniques were used: Lillie's oil red O method for the demonstration of lipids; the procedure of Tirmann and Schmelzer as given by Romeis (1948) for iron pigment; Lillie's allochrome or the van Gieson method for connective tissues.

The pituitaries were placed immediately after removal into weighed bottles containing 2 ml. of sublimate-formalin and stained by the methods given in a previous paper (Bielschowsky, 1953).

### RESULTS

### *The origin of the strain*

The strain designated NZY is now in the 29th generation. It originated in 1948 from a pair of mice with tan coat colour selected from the mixed colony kept in the Animal Department of the Medical School. One of the males of the 2nd generation of brother-sister mating was a piebald. He was mated to a sister with the original coat colour. In the 4th generation the first piebald female became available and was mated to a spotted brother. From that time only animals showing this character were used for propagation with the result that in the 7th and subsequent generations only piebald mice were born. Breast cancers appeared in breeding females of F10 and became more frequent in later generations. In the mixed mouse colony of the Medical School which maintains about 500 breeding females throughout the year breast cancers have been seen only twice during the last 5 years. However, most of the breeders are killed when less than 12 months old and nothing is known of the incidence of spontaneous tumours in older animals. Breast cancers do not occur in appreciable numbers in the other 4 inbred strains developed by us concurrently with the NZY. In fact, although many females completed their full life span, only one case of spontaneous mammary cancer has been found so far in the NZO and another in the NZC mice. A few appeared in old females of F14–15 but none in the following generations of the third strain; in the fourth this type of tumour has never been observed.

When complete autopsies of NZY mice were routinely performed it soon became evident that the great majority of females of the 18th and following generations had pituitaries which were either symmetrically or irregularly enlarged. We do not know when this tendency for pituitary enlargement appeared, it was first noticed in the 11th generation.

### *Some anatomical and physiological features*

Adult mice reach relatively high weights without being obese. The maximum weight of males is 42 g., that of breeding females 40 g. and of virgins 38 g. Values 3–5 g. lower are more common in our material and correspond to the usual weights of adult mice is the mixed colony from which the NZY mice originated. The animals are pink eyed. The pattern of white spotting varies considerably in extension and location. There is frequently a broad band of white hair encircling the lower back and abdomen. The fur covering the latter is generally of a lighter tan than that of the back. Deformities of the thorax are frequent and this strain appears to be more liable to infections of the lung than the other inbred mice kept in the department. However, losses have been light and the average life span has not been reduced significantly by such infections.

The females reach sexual maturity at an early age. The vagina opens between the 28th and 35th day and some mice become pregnant already at the age of 5 weeks. Vaginal smears taken for periods of 10 days in consecutive months revealed that regular 4–5 days cycles are rare in NZY virgins and that dioestrus lasts often for 7–9 or even more days. It seems, however, doubtful whether vaginal smears provide evidence that can be trusted entirely. We have observed more than once in stained sections of the vagina a squamous keratinizing epithelium covered by a layer of an exudate containing many leucocytes and desquamated cells. A viscous mucus-like fluid containing many polymorphs was frequently seen when taking smears.

In agreement with these observations is the irregular breeding performance. Some animals do not become pregnant when mated to males of proven fertility whereas others have their litters in rapid succession. Twelve of 91 animals belonging to F20–24 failed to become pregnant. Of the 79 fertile females 56 had a second, 29 a third and 21 a fourth litter. The number of new born mice in 62 first litters was 264. Of these 204 reached weaning age and the same rate of survival was found in second, third and fourth litters. The average litter-size was 4·2 for the first and slightly lower for subsequent pregnancies. It has to be stressed, however, that litter-size ranged from 1–12. The interval between the birth of two consecutive litters varied between 19 and 90 days. The females have a well-developed maternal instinct and even virgins 18 months of age will build a nest and care for newborn given to them.

*The sex organs and adrenals*

As already mentioned long periods of dioestrus occur frequently in young sexually mature females. In addition mucification of the vaginal epithelium was

---

EXPLANATION OF PLATES

FIG. 1.—Breast gland of virgin 3 months old. H. & E. × 18.
FIG. 2.—Breast gland of virgin 11 months old. H. & E. × 18.
FIG. 3.—Breast gland of virgin 20 months old. H. & E. × 18.
FIG. 4.—Macroscopically recognizable ducts. Breeder 16 months old.
FIG. 5.—Early intra-alveolar neoplastic lesion. Breeder 11 months old. H. & E. × 18.
FIG. 6.—Early intra-alveolar neoplastic lesion. Breeder 15 months old. H. & E. × 18.
FIG. 7.—Area of squamous metaplasia. Virgin 13 months old. H. & E. × 80.
FIG. 8.—Early adenoacanthomatous lesion. Breeder 15½ months old. H. & E. × 80.
FIG. 9—Keratin cyst from nipple area showing connection of the cyst with a mammary duct. Virgin 20 months old. H. & E. × 18.
FIG. 10.—Intratubular carcinoma. Breeder 21 months old. H. & E. × 80.
FIG. 11.—Intraductal papilloma. Breeder 11 months old. H. & E. × 18.
FIG. 12.—Intraductal papilloma. Breeder 21 months old. H. & E. × 80. (The lesions shown in Fig. 11 and 12 were found in breasts free of palpable tumours.)
FIG. 13.—Sebaceous lesion in mammary cancer. Breeder 10 months old. H. & E. ×113.
FIG. 14.—Sebum cells in midst of mammary cancer. Breeder 16 months old. H. & E. × 495.
FIG. 15.—Sebaceous adenoma of the skin. Breeder 17 months old. H. & E. × 27.
FIG. 16.—Sebaceous adenoma of skin in nipple area. Virgin 18 months old. H. & E. × 113.
FIG. 17.—Symmetrical enlargement of pituitary. Pituitary weight 7·0 mg. Virgin 8 months old, stilboestrol treatment for 6 months. Papanicolaou × 30.
FIG. 18.—Asymmetrical spontaneous enlargement of pituitary. Pituitary weight 5·2 mg. Papanicolaou × 30.
FIG. 19.—Typical picture of pituitary tumour. Pituitary weight 39·5 mg. Breeder 16 months old. Papanicolaou × 560.
FIG. 20.—Group of giant acidophils in pituitary tumour. Pituitary weight 3·5 mg. Breeder 11 months old. Papanicolaou × 560.

1

2

3

4

5

6

Bielschowsky, Bielschowsky and Lindsay.

7

8

9

10

11

12

13

14

15

16

Bielschowsky, Bielschowsky and Lindsay.

17

18

Bielschowsky, Bielschowsky and Lindsay.

19

20

Bielschowsky, Bielschowsky and Lindsay.

seen occasionally in stained sections. These observations motivated an inquiry into ovarian function and an attempt was made to induce deciduomas in normal virgins. For this purpose 20 mice, 95–110 days old, were used, 18 being in dioestrus and 2 in oestrus at the time of operation. When these animals were killed 4 days later deciduomas, 2 of macroscopic and one of microscopic size, were found in 3 mice. On histological examination 6 of the 18 animals which had been in dioestrus when operated upon showed marked mucification of the vagina and the deciduomas occurred in 3 of them. These findings indicate the presence of functionally active corpora lutea in unmated mice of the NZY strain. The ovaries contained 2 sets of corpora lutea, 10–12 of these structures being counted in sections from the area of maximum circumference. Large cells were present in many corpora lutea, especially in those from ovaries of mice with deciduomas or with mucification of the vagina. They did not quite reach the size of the lutein cells seen in lactating animals of similar age. The thyroids showed a normal picture, the colloid containing acini were lined by a cuboid epithelium. In the adrenal cortex a well-developed X-zone was seen only once and rests of this structure were still recognizable in a few animals. Small groups of the so-called A-cells of Woolley and Little (1945) were occasionally noted. The breast glands were remarkably well developed for virgins of this age (Fig. 1). There was a considerable variation in the number of alveoli present in the sections, in the degree of secretory activity as well as in the width of the ducts, some of which were definitely dilated.

In order to obtain some information on the endocrine status of females belonging to the age group in which mammary cancers begin to appear and the pituitaries to enlarge, 6 virgins, 6 breeders and 5 stilboestrol-treated females 8–11 months old were killed. The findings were compared with those obtained in 6 spayed animals 12–13 months of age. The ovaries of 11 intact untreated animals resembled those of 3–3½ months old mice, a picture which remains unchanged up to the age of 14 months. They contained follicles in all stages of development and multiple corpora lutea with rather large cells. Dividing lutein cells were often seen in recent corpora lutea. Only one of the virgins had ovaries free from corpora lutea. In this virgin the uterus weighed 185 mg. and the vagina was lined by a thick layer of squamous keratinizing epithelium, whereas in the other mice the weight of the uterus averaged 82 mg. and mucification of varying intensity was frequently present in the epithelial layer of the vagina.

The adrenals also differed little from those of the younger animals. The small subcapsular A-cells were present in larger numbers and reached deeper layers of the cortex. In 3 mice nodules of microscopic size, formed by large elements with foamy cytoplasm resembling the cells of the fascicularis were found in the adrenal cortex.

The mammary glands of the older females could easily be distinguished from those seen at 3–3½ months. Hyperplasia was already recognizable at autopsy. This increase in mammary gland tissue was due mainly to proliferation of alveoli (Fig. 2) which now began to form lobules. Pronounced secretory activity of the mammary epithelium and cystic dilatation of ducts was noted in 6 animals. These signs of enhanced functional activity were more marked in the breeders. Foci of lymphocytes and plasma cells, so-called inflammatory lesions, were present and the connective tissue had increased, especially around the ducts. These findings were common to virgins and mated animals, but in 3 of the breeders early neoplastic changes were also found. In the breast gland of an 11 months old mouse a small nodule just recognizable by naked eye inspection was noted at autopsy. This was found to be an intraductal papilloma (Fig. 11). In 2 other animals the histological examination revealed the presence of solid alveoli or tubules formed by an atypical mammary epithelium. We shall return to these neoplastic lesions when describing the mammary cancers.

During the second year of life the breast glands of virgins and breeders become increasingly hyperplastic. They are clearly recognizable at post mortem by their darker colour. Small cysts filled with a milky fluid were occasionally seen, but far more frequent were small nodules just visible to the naked eye. They were slightly harder to touch than the surrounding tissue. In 4 old breeders the ducts of one mammary gland were macroscopically recognizable as a black tree against the lighter background (Fig. 4). In other old animals diffuse areas of brownish discoloration were seen.

The first right or left breast gland which could be easily removed *in toto* was always taken for microscopic study in addition to any gland showing macroscopic abnormalities. The histological study of this material gave the impression of a continued growth process which affected ducts, alveoli and, to a lesser extent the connective tissue and did not abate in old age. A typical example of hyperplasia of the breast is depicted in Fig. 3 which shows a mammary gland of a 20-months-old virgin. In this animal the development of the breast had reached a degree comparable to that seen at the end of pregnancy. Functional activity varied from gland to gland and even in parts of the same breast, but in some animals nearly every lobe was secreting. Signs of senile involution were observed only in animals suffering from intercurrent disease.

The blackish discoloration seen at autopsy was found to be due to deposition of large amounts of haemosiderin in the epithelial cells lining the ducts and in macrophages nearby, whereas the brownish material filling the lumen failed to give an iron reaction. Routine sections taken from glands which at autopsy showed the picture of diffuse hyperplasia often revealed the presence of nodules of different kinds. Some consisted of groups of normal and/or atypical alveoli, some were areas of metaplasia and some were undoubtedly neoplastic lesions (Fig. 5, 6). The former have the same appearance as the so-called hyperplastic nodules found in most high breast cancer strains. The metaplastic lesions consisted of groups of alveoli lined by squamous, keratinizing epithelium (Fig. 7). Sometimes only one or two neighbouring alveoli were thus affected but often these lesions were more extensive. Around such squamous foci which commonly occur in virgins and breeders above the age of 14 months, lymphocytes had accumulated in large numbers and these changes were accompanied by an increase in connective tissue. In some of the macroscopically visible nodules similar metaplastic lesions were seen. Here the alveoli had become distended by masses of keratin and in most the adjacent alveoli were lined by an atypical high cuboid epithelium with strongly basophilic cytoplasm ; mitoses were frequent (Fig. 8).

Cystic lesions were found in the nipple area of 8 animals. They were lined by a squamous epithelium and filled with keratin. By serial sectioning it was possible to demonstrate a connection between these cysts and a mammary duct (Fig. 9).

In contrast to the progressive hyperplasia of the breast, the ovaries of aged animals show regressive changes. After the 14th month of life corpora lutea decrease in numbers, the lutein cells become smaller and finally even inactive corpora lutea are no longer present. Vagina and uterus show either the picture of unopposed oestrogenic stimulation or signs of beginning involution, the latter being more frequent in the very old animals.

Vagina, uterus and mammary glands of 5 of 6 mice ovariectomized at 6 weeks were found to be atrophic 12 months later. The uterus of the sixth weighed 47 mg. as compared with a mean of 16 mg. in the others. The adrenals showed a higher degree of nodular hyperplasia than is commonly seen in intact females of similar age. So far we have not observed in old spayed females hyperplasia of breast glands or mammary tumours.

*The mammary cancers*

Table I gives the incidence of palpable mammary cancers in females older than 6½ months, the age at which the earliest breast cancer was discovered in a breeder. In virgins no breast tumours were found before the 14th month. Of the 31 unmated females available for study 20 were 13–22 months old when killed and 7 of these had one or more mammary cancers. In 49 of the 84 breeders breast tumours developed. If any conclusions can be drawn from these small numbers, it seems that in virgins breast cancers develop relatively late in life and that their incidence rises with advancing age. In breeders breast cancers develop earlier and the incidence diminishes after the age of 17 months. There was no evidence for an increased frequency of mammary cancers in certain families and no relationship could be established between number of pregnancies and incidence of breast cancers. Of 7 mated animals without offspring 3 had palpable breast tumours and a 4th an early adenoacanthoma. If all the neoplastic lesions had been counted which were discovered either at autopsy where they presented themselves as small nodules, or at microscopic examination of breast glands, the incidence of breast tumours would be considerably higher. By excluding the non-palpable lesions we have eliminated those whose fate was uncertain. In our experience nearly all the palpable tumours grow progressively, although there is good reason to believe that not all the neoplastic lesions progress to malignancy. Regression of a palpable tumour was observed only once. This occurred in a nursing animal.

TABLE I.—*Incidence of Breast Tumours in Virgins and Breeders*

| | Virgins | | | Breeders | | |
|---|---|---|---|---|---|---|
| Age group (months) | 6–12 | 12–18 | 18–22 | 6–12 | 12–18 | 18–23 |
| Total number | 11 | 13 | 7 | 21 | 49 | 14 |
| Mice with tumours | 0 | 3 | 4 | 14 | 32 | 3 |

Of the 56 mice with breast cancers 50 had 1, 5 had 2 and 1 had 3 palpable tumours. These numbers probably do not reflect the true incidence of multiple mammary cancers since most mice were killed soon after a subcutaneous mass was found. In 6 instances the interval between discovery of a breast tumour and autopsy was 5–8 weeks and still no other palpable tumour appeared. Distant metastases were found in 4 animals, three times in the lung and once in the liver.

*The morphology of the breast tumours*

Of the 63 palpable tumours available for classification 35 had a predominantly alveolar structure (Group A and B of Andervont and Dunn, 1950). In 9 of these some secretory activity was noted, and in 3 others small foci formed by squamous keratinizing epithelial cells were present. In 7 breast tumours the metaplastic changes were so extensive as to justify the diagnosis of adenoacanthoma. In the remaining 19 carcinomas an alveolar structure could not be found or was only occasionally recognizable. In 8 of these tumours the epithelial cells grew in the form of solid tubules surrounded by a delicate stroma. A well-differentiated specimen of such an intratubular carcinoma (Bonser, 1954) is shown in Fig. 10. Some neoplasms were quite undifferentiated and two, formed by detached cells, resembled closely the tumour depicted by Andervont and Dunn (1950) in their Fig. 6. Two of the breast tumours were sarcomas, one of which contained a few nests of atypical epithelial cells. In 6 animals intraductal papillomas were found (Fig. 11, 12). They occurred in glands not affected by a palpable tumour.

Quite unusual was the presence of sebaceous elements in 4 cancers of the breast. As seen in Fig. 13 islands of hyperplastic sebaceous glands were surrounded by cords of typical mammary cancer cells. Blind ending ducts lined by a stratified squamous epithelium were present but no hair follicles were found in these lesions. In one case it was possible to measure the distance between epidermis and an accumulation of sebaceous glands, it was found to be 2·5 mm. Sebaceous cells were present not only as distinct adenoma-like structures, but twice also in the form of small groups in the midst of mammary cancer cells, as if these elements had differentiated into sebum cells (Fig. 14).

*Sebaceous adenomas of the skin*

The sebaceous lesions found in sections of otherwise typical breast tumours should be distinguished from sebaceous adenomas of the skin, 6 of which were found in females 12–20 months old. They were situated between epidermis and panniculus carnosus (Fig. 15). All were benign, slow growing and one underwent cystic degeneration. They arose in different areas of the skin, 2 were found near normal nipples (Fig. 16) and 1 in the perineum.

*The pituitary*

All the 40 untreated males, the oldest of which reached the age of 24 months, had normal pituitaries (maximum weight 2·2 mg.) but in the females pituitary weight varied with age.

In intact females the pituitary weights are normal at the age of $3\frac{1}{2}$ months, increased at the age of 8–11 months; gonadectomy early in life prevents this enlargement of the gland. When a pellet weighing approximately 5 mg. and consisting of 97·5 per cent cholesterol and 2·5 per cent stilboestrol was implanted into normal males or females 4–5 weeks old their pituitaries were found to be grossly enlarged 7–8 months later. This weight increase was most striking in the males. These results are summarized in Table II.

Histologically the adenohypophysis of intact 3–$3\frac{1}{2}$-months-old virgins showed a normal distribution of chromophil and chromophobic elements. At the age of 8–11 months however, the anterior lobes of intact untreated females contained areas, mostly situated near the posterior border and in the most lateral part of the adenohypophysis, in which fully granulated acidophils were reduced in numbers. In these regions a partially or totally degranulated cell with a large Golgi body predominated. Since all transitions from fully granulated acidophils to completely degranulated forms were present we do not doubt that the latter

TABLE II.—*Pituitary Weights of Normal and Spayed Females and of Stilboestrol-treated Animals*

| Virgins | | | | Breeders | | Spayed female. | | Stilboestrol-treated Female. | | Stilboestrol-treated Male. | |
|---|---|---|---|---|---|---|---|---|---|---|---|
| Age (months) | mg. | Age (months) | mg. | Age (months) | mg. | Age (months) | mg.. | Age (months) | mg. | Age (months) | mg |
| 3½ | 1·7 | 8½ | 3·4 | 8 | 3·2 | 12 | 1·3 | 8½ | 5·6 | 8 | 17·1 |
| 3½ | 1·8 | 10½ | 3·4 | 10 | 3·2 | 12 | 1·3 | 9 | 6·0 | 8½ | 20·0 |
| 3½ | 2·0 | 11 | 2·8 | 11 | 3·0 | 12½ | 1·6 | 9 | 6·3 | 9 | 17·8 |
| 3½ | 2·0 | 11 | 3·0 | 11 | 3·2 | 12½ | 2·0 | 9 | 7·0 | 9 | 18·7 |
| 3½ | 2·1 | 11 | 3·1 | 11 | 3·4 | 13 | 1·3 | 9 | 7·2 | 9 | 20·2 |
| 3½ | 2·2 | 11 | 4·0 | 11 | 3·6 | 13 | 1·3 | — | — | 10 | 22·4 |

are derived from the acidophilic cells with enlarged Golgi apparatus. A moderate number of mitoses was also found in these areas and it was often possible to identify the dividing cells as acidophils by their granulation. At this stage only a very mild hyperaemia was noticeable so that the gain in weight of the pituitary which takes place between the 4th and 8th month of life must be attributed mainly to an increase in acidophils and chromophobic cells derived from them. These changes were entirely absent from the pituitaries of spayed animals and much exaggerated in those of stilboestrol-treated virgins. A typical example of a symmetrically enlarged pituitary of a stilboestrol-treated female is depicted in Fig. 17. Here nearly half of the anterior lobe was free of normal, fully granulated acidophils. From the results given in Table II it is obvious that pituitary enlargement occurs only in the presence of the ovaries or after treatment with oestrogen. To judge from the atrophy of the secondary sex organs seen one year after ovariectomy, it seems that the amount of estrogen secreted by the adrenals is negligible and that the ovaries are the major source of oestrogen in this strain. The pronounced enlargement of the pituitaries of the males treated with stilboestrol leaves no doubt that in the NZY strain this gland is highly susceptible to oestrogen.

With progressing age the pituitaries of intact, untreated NZY females tend to increase in size and become more hyperaemic. Table III gives the range of pituitary weights of 105 untreated females 6½–24 months old. Only 19 animals had pituitaries weighing less than 3 mg. and the largest pituitaries were found in aged breeders. Whereas during the first year of life most adenohypophyses are symmetrical, in the second the gland tends to become nodular and haemorrhagic areas appear. Nodularity is not restricted to the very large glands weighing more than 10 mg. but can occasionally be observed in pituitaries 3·0–5 mg. In such glands one half of the anterior lobe often contains a slightly raised area which is darker than the surrounding tissue. With increasing pituitary size

TABLE III.—*Pituitary Weights of Females* 6–24 *Months of Age*

| Age | Number of mice | Number of animals with pituitary weights of –3 mg. | 3–5 mg. | 5–10 mg. | 10–30 mg. | 30–50 mg. |
|---|---|---|---|---|---|---|
| 6–12 | Virgins : 10 | 6 | 3 | 1 | — | — |
| | Breeders : 18 | 6 | 9 | 3 | — | — |
| 12–18 | Virgins : 14 | 3 | 7 | 4 | — | — |
| | Breeders : 44 | 3 | 13 | 14 | 12 | 2 |
| 18–24 | Virgins : 6 | — | 3 | 3 | — | — |
| | Breeders : 13 | 1 | 1 | 4 | 5 | 2 |

the nodules become more numerous and the largest glands appear frankly tumorous. Such pituitaries produce signs of increased intracranial pressure. Invasion of the brain or other adjacent structures was never observed.

Histologically the largest pituitary " tumours " did not differ in any way from the smaller lesions just recognizable at autopsy except for increased vascularity and tendency for haemorrhages. Fig. 18 shows an early nodule in an asymmetrically enlarged gland and the reduction of the acidophils in one half of the anterior lobe. There is no need for a detailed description of the cytology of these pituitary growths. They resemble closely those seen in mice or rats treated with oestrogen or the spontaneous acidophilic tumours of the rat which secrete prolactin. The typical elements of these growths are cells with a large Golgi apparatus. Some contain acidophilic granules whereas others are free of them, the latter being more numerous. There is some variation in size and shape of the cells forming the nodules and mitoses can be found in moderate numbers. A section from a typical lesion is shown in Fig. 19. One additional feature is worth mentioning : the presence of foci of abnormally large acidophils with well-recognizable Golgi apparatus in areas which otherwise show the picture described above (Fig. 20). On morphological grounds one has to assume that the cells with the large Golgi apparatus and the tendency to lose their acidophilic granules, whether they occur in areas of simple hyperplasia or in adenomas, are actively secreting cells.

All animals in which a breast cancer developed in the second year of life had pituitaries weighing more than 3 mg. but in 15 females in which the pituitaries weighed from 5·1 to 51·2 mg. no breast cancer was found.

*Other tumours in NZY mice*

In the females, apart from breast and pituitary, the lung was the organ most frequently affected by neoplasia. All of the 20 nodules found were benign adenomas. In addition 3 granulosa cell tumours of the ovary, 2 haemangio-endotheliomas of the uterus, 2 leukaemias, 1 acanthotic papilloma of the skin and 1 $\beta$-cell adenoma of the pancreas were seen. In the 40 males 3 adenomas and 2 cancers of the lung, 2 sarcomas, 1 benign hepatoma and 1 leukaemia were found.

### DISCUSSION

The enormous literature on mammary cancer in mice has recently been reviewed by Gardner (1953), Dmochowski (1953) and Lacassagne (1955). We have therefore limited ourselves to quote a few papers dealing mainly with the role of hormones in their development.

The mammary glands of NZY females become increasingly hyperplastic with advancing age and do not undergo senile involution. The rate of mammary growth is more rapid in breeders in which mammary cancers appear at an earlier age and in larger numbers. The adenohypophysis too increases progressively in size, and the largest glands have been found in mated females. The mammary and pituitary hyperplasias and the supervening neoplastic changes depend on the presence of the ovaries. That oestrogen is the ovarian factor responsible for the hyperplasia of the pituitary is most likely since in both sexes stilboestrol enlarges this gland. Proliferation of one type of functionally active acidophils seems to account for most of the " spontaneous " enlargement of the anterior lobe. We have mentioned the similarity of these elements with the cells found in the pituitaries of rats and mice subjected to stimulation by moderate doses of oestrogen. Whereas most authors have described these lesions as chromophobic adenomas, we are of the opinion that the prevailing element is a more or less degranulated acidophil. We have classified the nodular lesions as adenomas because they were too pleomorphic to be considered as nodular hyperplasias. It is not yet known at which stage of development they become independent of ovarian stimulation. Finally we wish to stress that the pituitary lesions described in this paper are quite different from those observed by Dickie and Woolley (1949) who found the following sequence in mice gonadectomized shortly after birth: first adrenal cortical tumours appeared and later basophilic adenomas of the pituitary concurring with marked stimulation of the mammary glands.

Meites and Turner (1942) and Chamorro (1952) have shown that mammogenic potency increases in the pituitaries of rodents treated with oestrogen. Lyons and his collaborators (1953, 1955) have clearly demonstrated the importance of somatotrophin and of prolactin for mammary growth. These two hormones are secreted by acidophils. The fact that the NZY females are not larger than the males militates strongly against growth hormone being secreted by the pituitary cells which increase progressively in numbers and which form the adenomas in the anterior lobe. Mammogenic acidophilic tumours of the pituitary of the rat have been described by Lacour (1950) and by Bielschowsky (1954). Mühlbock (1953) has found spontaneous prolactin secreting tumours in mice and Furth (1955) has obtained transplantable pituitary tumours with mammogenic activity (Upton and Furth, 1955).

Prolactin has not only mammotrophic but also luteotrophic action. In most strains functionally active corpora lutea are present only during pregnancy and lactation; but in NZY mice they must occur outside periods of gestation. Otherwise it would not have been possible to induce in young virgins deciduomas by traumatization of the uterus. Prolonged periods of dioestrus, mucification of the vagina and corpora lutea formed by large lutein cells, frequently found in females up to the age of 14 months, are evidence in favour of a relatively high progesterone secretion in NZY females.

It seems that the unusual degree of mammary development seen during the second year of life is due to the combined action of pituitary and ovarian factors. Loeb and Kirtz (1939) showed that transplanted anterior lobes of the pituitary stimulated the mammary glands and favoured the preservation of corpora lutea. The incidence of mammary cancers in virgin A mice treated with pituitary transplants rose above that of intact A breeders. More recently Mühlbock (1955) using the same procedure obtained breast tumours in virgins of 3 strains which did not possess the milk factor. In only one of these was it possible to obtain breast tumours by the administration of oestrogens. The importance of active corpora lutea in the genesis of mammary cancer in mice was clearly demonstrated by Law (1941). Symeonidis (1945) and Trentin (quoted from Gardner, 1953) demonstrated that progesterone favoured the development of spontaneous mammary cancers in this species. Recently Jull (1954) showed that the number of mammary cancers induced by a limited dose of methylcholanthrene was considerably higher in animals treated with a combination of oestrogen and progesterone than in mice injected with oestrogen only.

The breast tumours found in NZY mice show perhaps more variation in structure than is commonly seen in high mammary cancer strains carrying the milk factor. In the NZY females most of them arise in alveoli in the manner described by Bonser (1945), but neoplastic changes in the ducts are by no means rare. Often it appeared as if all epithelial elements in one field underwent neoplastic changes at the same time. Since adenoacanthomas were decisively less frequent than the small squamous metaplastic nodules discovered at autopsy it seems that only few of them progressed to the state of malignancy. Pullinger (1949, 1954, 1955) has given a detailed account of the morphology and the nature of the metaplastic lesions found in the breasts of RIII mice.

We are not yet in the possession of all the information necessary for a complete analysis of the factors instrumental in the genesis of mammary cancers in NZY females. The possible role of the milk factor is the main problem still to be solved. So far mammary cancers have failed to appear in female offspring of NZY females mated to males belonging to a low cancer strain (NZC). The hybrids have now reached an age at which breast tumours were frequent in their mothers. The presence of multiple hyperplastic nodules in the breast glands of breeders below the age of 12 months suggests that the NZY strain might carry the milk factor. However, in view of the striking particularities of NZY females in respect to pituitary and ovarian function, we are reluctant to consider these hyperplastic nodules as pathognomonic for the Bittner agent.

Of special interest were the sebaceous lesions which so far have been found exclusively in females. The susceptibility of sebaceous glands to hormones has been demonstrated by several authors. Haskin, Lasher and Rothman (1953) obtained hyperplasia of sebaceous glands in rats treated with progesterone, an effect not observed by Ebling (1947). Carlisle (1954) studied the reaction of the sebaceous glands situated in the nipple area of rabbits to daily injections of oestrone and progesterone followed by luteotrophin. Under this treatment the sebaceous glands enlarged, became multilobulated and finally gave rise to new mammary ducts. It seems therefore that in rodents an intimate relationship exists between sebaceous and mammary glands which might explain the genesis of the sebaceous lesions in abnormal mammary glands of NZY females. Chemically induced sebaceous gland tumours of the skin of mice have been observed by Twort and Twort (1930), Twort and Bottomley (1932) and recently in " Crew " mice by Rous (1956). Spontaneous sebaceous adenomas seem to be extremely rare in mice. Further investigations will be required to elucidate the role of hormonal factors in the pathogenesis of these skin tumours.

### SUMMARY

An account of the origin of a new strain of mice has been given.

At present the incidence of mammary cancer in this strain is 58 per cent for breeders above the age of $6\frac{1}{2}$ months and 35 per cent for virgins in the second year of life.

More than 80 per cent of intact females above the age of 6 months have enlarged pituitaries, but the pituitary weights of males and of gonadectomized animals are normal.

The mammary glands reach an unusual degree of development in virgins as well as in breeders. The excessive stimulation of the mammary gland is considered to be due in part to an elevated secretion of ovarian hormones and in part to the mammogenic action of prolactin.

The induction of deciduomas in young virgin mice suggests secretion of progesterone by functionally active corpora lutea.

Sebaceous adenomas of the skin and sebaceous lesions in mammary cancers have been found in this strain.

### REFERENCES

ANDERVONT, H. B. AND DUNN, T. B.—(1950) *J. nat. Cancer Inst.*, **10**, 895.
BIELSCHOWSKY, F.—(1953) *Brit. J. Cancer*, **7**, 203.—(1954) *Ibid.*, **8**, 154.
BIELSCHOWSKY, M. AND BIELSCHOWSKY, F.—(1956) *Aust. J. exp. Biol. med. Sci.*, **34**, 181.
BONSER, G. M.—(1945) *J. Path. Bact.*, **57**, 413.—(1954) *Ibid.*, **68**, 531.
CARLISLE, D. B.—(1954) *Quart. J. micr. Sci.*, **95**, Part I, 79.
CHAMORRO, A.—(1952) *Ciba Foundation Colloquia on Endocrinology*, **1**, 87.
COPPEDGE, R. L. AND SEGALOFF, A.—(1951) *J. clin. Endocrin.*, **11**, 465.
DICKIE, M. M. AND WOOLLEY, G. W.—(1949) *Cancer Res.*, **9**, 372.
DMOCHOWSKI, L.—(1953) *Advanc. Cancer Res.*, **1**, 104.
EBLING, F. J.—(1947) *J. Endocrin.*, **5**, 297.
FURTH, J.—(1955) ‘ Recent Progress in Hormone Research ’, **11**, 221.
GARDNER, W. U.—(1953) *Advanc. Cancer Res.*, **1**, 173.
HADFIELD, G.—(1956*a*) *Brit. med. J.*, i, 94.—(1956*b*) *Ibid.*, i, 1507.
HASKIN, D., LASHER, N. AND ROTHMAN, S.—(1953) *J. invest. Derm.*, **20**, 207.
JULL, J. W.—(1954) *J. Path. Bact.*, **68**, 547.
LACASSAGNE, A.—(1955) *J. Endocrin.*, **13**, IX.
LACOUR, F.—(1950) *C.R. Soc. Biol. Paris*, **144**, 248.
LAW, L. W.—(1941) *Proc. Soc. exp. Biol. N.Y.*, **48**, 486.
LOEB, L. AND KIRTZ., M. M.—(1939) *Amer. J. Cancer*, **36**, 56.
LYONS, W. R., LI, C. H., COLE, R. D. AND JOHNSON, R. E.—(1953) *J. clin. Endocrin. Metab.*, **13**, 836.
*Idem*, LI, C. H., JOHNSON, R. E. AND COLE, R. D.—(1955) *Proc. International Symposium on Hypophyseal Growth Hormone, Nature and Actions.* New York (Blakiston Comp. Inc.), 461.
MEITES, J. AND TURNER, C. W.—(1942) *Endocrinology*, **30**, 711.
MÜHLBOCK, O.—(1953) ‘ Derde Jaarboek van Kankeronderzoek en Kankerbestrijding in Nederland ’, 14.—(1955) *Strahlentherapie*, **96**, 274.
PULLINGER, B. D.—(1949) *Brit. J. Cancer*, **3**, 494.—(1954) *Ibid.*, **8**, 161.—(1955) *Ibid.*, **9**, 613.
ROMEIS, B.—(1948) ‘ Mikroskopische Technik ’, München, (Leibniz Verlag), 282.
ROUS, P.—(1956) *Proc. Amer. Ass. Cancer Res.*, **2**, 143.
SCOWEN, E. F. AND HADFIELD, G.—(1955) *Cancer*, **8**, 890.
SEGALOFF, A., GORDON, D., HORWITT, B. N., SCHLOSSER, J. V. AND MURISON, P. J.—(1951) *Ibid.*, **4**, 319.
SYMEONIDIS, A.—(1948) *Acta Un. int. Cancr.*, **6**, 163.
TRENTIN, J. J., quoted from Gardner, W. U.—(1953) *Advanc. Cancer Res.*, **1**, 217.
TWORT, C. C. AND BOTTOMLEY, A. C.—(1932) *Lancet*, ii, 776.
*Idem* AND TWORT, J. M.—(1930) *Ibid.*, i, 1331.
UPTON, A. C. AND FURTH, J.—(1955) *J. nat. Cancer Inst.*, **15**, 1005.
WOOLLEY, G. W. AND LITTLE, C. C.—(1945) *Cancer Res.*, **5**, 193.